# The impact of basic psychological needs on exercise behavior among college students: the chain mediating effects of self-efficacy and sports motivation

**DOI:** 10.3389/fpsyg.2026.1774246

**Published:** 2026-04-24

**Authors:** Xuening Li, Wei Xu, Zhenzhong Du, Huasen Yu, Jing Wang

**Affiliations:** 1 Department of Physical Education, Nanjing University of Posts and Telecommunications, Nanjing, China; 2 College of Physical Education and Health, East China Normal University, Shanghai, China; 3 College of Physical Education, Dalian University, Dalian, China

**Keywords:** basic psychological needs, exercise behavior, mediating, self-efficacy, sports motivation

## Abstract

**Introduction:**

Prior research has suggested that basic psychological needs are closely associated with exercise behavior. Building on this perspective, the present study sought to examine the relationships among basic psychological needs, exercise behavior, sports motivation, and self-efficacy within a multivariate framework.

**Methods:**

A cross-sectional survey was performed among 1,056 college students, with a balanced gender distribution (50.66% male, 49.34% female). Structural equation model (SEM) analysis was conducted using Amos 26.0 to test the associations among the study variables and to test potential mediating pathways.

**Results:**

The SEM results revealed that basic psychological needs were positively associated with and exercise behavior. Upon integrating sports motivation and self-efficacy into the model, both direct and indirect associations were observed. Specifically, basic psychological needs were indirectly associated with exercise behavior through sports motivation and self-efficacy. Additionally, a sequential mediation pattern was supported, whereby basic psychological needs were associated with sports motivation, which was in turn associated with self-efficacy, and subsequently related to exercise behavior.

**Conclusion:**

These findings provide evidence of the complex associations among basic psychological needs, exercise behavior, sports motivation, and self-efficacy. However, given the cross-sectional design, the observed relationships should be interpreted with caution, and no causal inferences can be drawn. Future longitudinal or experimental studies are needed to further clarify the directionality of these relationships. This pattern underscores the potential relevance of motivational and cognitive factors in understanding individual differences in physical activity engagement among college students.

## Introduction

Health challenges facing adolescents have become increasingly severe worldwide (Sawyer et al., 2012). According to the World Health Organization (2020), nearly 81% of adolescents globally exhibit varying degrees of physical health decline, with approximately one-fifth being overweight or obese. Similarly, findings from the 2019 Chinese Students’ Physical Fitness and Health Survey indicate that current efforts to curb obesity among college students have yielded limited success. A substantial body of evidence demonstrates that regular physical activity is associated with enhanced physical and mental health, better sleep quality, reduced stress and mood disorders, and improved brain and bone development, while being linked to a lower risk of chronic diseases (Chow et al., 2022; Varanoske et al., 2022). Nevertheless, persistently low levels of physical activity, together with insufficient positive exercise behaviors and awareness, remain a major public health concern (Van Sluijs et al., 2007). Therefore, promoting exercise behavior is crucial for the healthy development of college students.

Individuals’ exercise behavior is influenced by a complex interplay of demographic, economic, social and psychological factors (Wang et al., 2022). Self-Determination Theory (SDT) provides a comprehensive framework for explaining self-determined human behavior (Conner and Norman, 2015), positing that the satisfaction of basic psychological needs fosters intrinsic motivation, which in turn exerts a positive influence on goal pursuit and behavioral engagement. Within this framework, basic psychological needs comprise three core dimensions: autonomy (the will and willingness to engage in an activity), competence (an individual’s perceived ability to perform an activity), and relatedness (the sense of caring and belonging that is gained through connecting with close others and feeling important to a social group) (Ryan and Deci, 2017; Ryan et al., 2009). With the development of positive psychology, an expanding body of research has examined the role of basic psychological needs in promoting exercise behavior (Wierts et al., 2024). Evidence indicates that the satisfaction of these needs positively associated with perceived exercise benefits and provides strongly support for the long-term effectiveness of physical activity participation (Kang et al., 2020). Moreover, higher levels of psychological need satisfaction are consistently associated with increased exercise engagement among adolescents (Sylvester et al., 2018). Despite substantial evidence supporting a positive relationship between basic psychological needs and adolescent exercise behavior, the psychological mechanisms underlying this association remain insufficiently understood. Therefore, elucidating the relationship between basic psychological needs and exercise behavior is of critical theoretical and practical significance.

Research suggests that self-efficacy may be associated with both basic psychological needs and exercise behavior (Wang et al., 2022). Self-efficacy refers to an individual’s belief in their capability to organize and execute actions required to achieve specific goals and is closely related to perceived competence and self-confidence (Bandura, 1993). Accumulating evidence indicates that the degree of basic psychological needs plays a critical role in self-efficacy development. Specifically, the satisfaction of autonomy needs has been found to be positively associated with self-efficacy, as individuals who experience greater volitional control tend to perceive themselves as more capable in their actions (Meng, 2022). Competence satisfaction has been positively associated with self-efficacy, reflecting that individuals who report higher levels of competence satisfaction also tend to report higher perceived capability (Ümmet, 2017). Additionally, Bandura (1977) argued that self-efficacy shapes behavioral enactment through its influence on cognitive mediating processes. Within the sport and exercise domain, self-efficacy has been consistently identified as a robust determinant of both exercise participation and adherence. Individuals with higher self-efficacy are more inclined to set challenging goals, maintain regular engagement in physical activity, and persist in the face of obstacles and setbacks (Samson and Solmon, 2011). Studies have demonstrated that self-efficacy is positively associated with physical activity frequency (McAuley et al., 2000) and significantly correlated with its duration (Lim et al., 2005). Taken together, these findings suggest that basic psychological needs may be indirectly associated with exercise behavior by self-efficacy.

Emerging evidence indicates that sports motivation may be may be associated with both basic psychological needs and exercise behavior (Li et al., 2024). As highlighted by SDT, satisfaction of basic psychological needs is associated with higher levels of intrinsic motivation, which is, in turn associated with greater exercise participation and adherence (Ryan and Deci, 2017). Intrinsic motivation arises from inherent interest and enjoyment in the activity itself and is generally more enduring and stable than externally regulated forms of motivation (Ingledew and Markland, 2008). For example, individuals in autonomy-supportive environments tend to report stronger autonomous motivation, which is associated with exercise commitment and performance outcomes (Cai, 2016). In addition, self-efficacy may be associated with both sports motivation and exercise behavior. Although self-efficacy is traditionally viewed as an antecedent of motivation (Bandura, 1977), emerging evidence suggests that the association between these constructs may be reciprocal and context-dependent (Tokarska and Rogowska, 2025). According to SDT framework (Ryan and Deci, 2000a), higher levels of autonomous motivation can promote sustained behavioral engagement, thereby increasing opportunities for mastery experiences, which are a primary source of self-efficacy. In this sense, sports motivation may operate as an upstream driver that indirectly contributes to the development of self-efficacy through enhanced participation and successful performance experiences. Empirically, recent research has demonstrated a close relationship between self-efficacy and sports motivation (Tao et al., 2024). For instance, Neace et al. (2022) reported that higher levels of motivation are associated with higher self-efficacy for engaging in physical activity, while Li et al. (2023) found a significant association between motivation and self-efficacy. Consistent with prior findings, sports motivation contributes to enhanced self-efficacy, which in turn positively correlates with increased physical activity engagement (Klompstra et al., 2018; Zhao et al., 2023). Collectively, these findings support a theoretically plausible pathway in which sports motivation contributes to self-efficacy, which subsequently promotes exercise behavior.

As previously noted, while existing research has established positive associations between basic psychological needs, exercise behavior, sports motivation, and self-efficacy, most prior studies a lack of focus on the sequential chain mechanism among these variables and a tendency to examine them in isolation or through simple single-mediation configurations. In contrast, the present study proposes a comprehensive chain mediation model that highlights the sequential mechanism, providing a more comprehensive and nuanced understanding of the psychological mechanisms than previous studies that only examined partial relationships. By uncovering these cascading mechanisms, our findings will offer a robust theoretical foundation for educators and policymakers to design targeted interventions aimed at fostering college students’ exercise behavior. Based on the above theoretical elaboration and research design, we propose the following hypotheses: (H1) Basic psychological needs are positively associated with exercise behavior; (H2) basic psychological needs are positively associated with exercise behavior through self-efficacy; (H3) basic psychological needs are positively associated with exercise behavior via sports motivation; and (H4) basic psychological needs are positively associated with exercise behavior through the sequential associations of sports motivation and self-efficacy.

## Method

### Participants and procedure

From Oct 4th to Oct 11th, 2024, a simplified cluster sampling method was adopted to randomly recruit participants from three major universities across Shandong, Liaoning, and Jilin provinces in China. Specifically, two universities were randomly selected within each of these regions. Data collection for the study was conducted via an online questionnaire administered on the “Wenjuanxing” platform. A total of 1,056 college students were included in the subsequent analysis after removing 32 invalid responses, which consisted of regular answering patterns or faulty data, yielding an effective response rate of 96.95%. The participants ranged from 17 to 24 years old, with an average age of 19.99 years and a standard deviation of 1.73. There was a relatively balanced gender proportion, with male comprising 50.66% and females 49.34% of the sample.

This study received approval from the East China Normal University Committee on Human Research Protection (HR 284–2024). Prior to study participation, all participants were provided with a comprehensive explanation of the research objectives, and were explicitly informed that their responses would be treated with strict confidentiality and anonymity. Written informed consent was obtained electronically from all participants. Before completing the online questionnaire, participants were fully informed about the purpose, procedure, anonymity, confidentiality, and voluntary nature of the study. Only participants who actively indicated their informed consent by checking the agreement statement could proceed to the formal survey. The study was conducted in strict adherence to the revised ethical principles stipulated in the Declaration of Helsinki.

### Measures

#### Rating Scale of Physical Activity (PARS-3)

A Chinese edition of Rating Scale of Physical activity (PARS-3), introduced and revised by Liang (1994), was used to assess the exercise behavior. The questionnaire’s author has made it freely accessible to the public. The scale consists of three items, evaluating time, frequency, and intensity of physical activity. Physical Activity = time scores × frequency scores × intensity scores. The intensity and frequency of physical exercise are scored from 1 to 5, and time is rated from 0 to 4. The total score ranges from 0 to 100 points. The corresponding criteria for physical activity levels are: Low exercise ≤ 19 points, moderate exercise 20 to 42 points, vigorous ≥ 43 points. In this study, the internal consistency reliability of the GSES was examined using Cronbach’s alpha and CR, while the convergent validity was assessed using factor loadings and AVE (Fornell and Larcker, 1981). The results indicated strong reliability (*α* = 0.603; CR = 0.78). Additionally, all factor loadings ranged from 0.58 to 0.61, and the AVE value was 0.52. These results show the good reliability and validity of the scale the Cronbach’s alpha coefficient was 0.603.

#### Basic Psychological Needs in Exercise Scale (BPNES)

The basic psychological needs were assessed using Basic Psychological Needs in Exercise Scale (BPNES) developed by Vlachopoulos and Michailidou (2006), and the Chinese version of the BPNES translated by Liu et al. (2013). This scale consists of 12 items and contains three subscales including competence, relatedness, and autonomy. The scale is scored on a 7-point scale ranging from 1 (strongly disagree) to 7 (strongly agree), with higher scores indicating a higher level of need satisfaction for the participants. First, we used Cronbach’s alpha and composite reliability (CR) to assess Internal consistency reliability of BPNES. The overall Cronbach’s alpha was 0.953, and the alphas for the autonomy, relatedness, and competence subscales were 0.909, 0.904, and 0.876, respectively. The CR values for competence (0.91), relatedness (0.91), and autonomy (0.88) all exceeded the recommended threshold of 0.70. Second, we used the factor loadings and average variance extracted (AVE) to evaluate convergent validity. Confirmatory factor analysis supported the scale structure (χ^2^/df = 6.71, GFI = 0.96, NFI = 0.98, IFI = 0.98, CFI = 0.98, RMSEA = 0.074), with all factor loadings ranging from 0.75 to 0.91. The AVE values for competence, relatedness, and autonomy were 0.72, 0.71, and 0.64, respectively, all above the cutoff value of 0.50. Overall, these results demonstrate the good reliability and validity of the BPNES.

#### The General Self-Efficacy Scale (GSES)

The Chinese version of the General Self-Efficacy Scale (GSES) was employed to measure individual self-efficacy levels in this study (Zhang and Schwarzer, 1995). The scale consists of 10 items, scored on a 4-point scale ranging from 1 (not at all true) to 4 (exactly true). Higher total scores indicate higher levels of self-efficacy in individuals. The scale has been widely used in research involving university students, showing good internal consistency (Li et al., 2024). In the present study, the internal consistency reliability of the GSES was evaluated using Cronbach’s alpha and CR, while the convergent validity was examined using factor loadings and AVE. The results indicated strong reliability (*α* = 0.926; CR = 0.93). Confirmatory factor analysis supported the scale structure (χ^2^/df = 7.55, GFI = 0.96, NFI = 0.97, IFI = 0.97, CFI = 0.97, RMSEA = 0.079), with factor loadings ranging from 0.52 to 0.85 for all the items. The AVE value (0.57) exceeded the recommended threshold. Overall, these results demonstrate satisfactory reliability and validity of the scale.

#### The Sport Motivation Scale (SMS)

The Sport Motivation Scale, developed by French scholar Briere et al. (1995) and translated into Chinese by Peng (2012), was used to measure participants’ levels of sport motivation. The Chinese version of sports motivation scale is available and free for use. This scale includes 28 items and comprises three components: intrinsic motivation, extrinsic motivation, and amotivation. Each item is scored on a 7-point Likert scale ranging from 1 (completely disagree) to 7 (completely agree). Higher total scores indicated higher levels of sport motivation. However, one item (“To show people that I’m good at this sport”) on the optimistic scale had a factor loading of less than 0.40 (Ford et al., 1986) and was removed from the analysis. The scale has been widely used in motivation-related research in the field of sports (Wang, 2021). First, internal consistency of the SMS was assessed using Cronbach’s alpha and CR. The overall Cronbach’s alpha was 0.962, and the alphas for the intrinsic motivation, extrinsic motivation, and amotivation subscales were 0.951, 0.921, and 0.733, respectively. The CR values for intrinsic motivation (0.95), extrinsic motivation (0.93), and a motivation (0.83) were over the recommended threshold of 0.70, indicating good reliability. Second, convergent validity was evaluated using factor loadings and AVE. Confirmatory factor analysis of the scale supported the scale structure (χ^2^/df = 6.45, GFI = 0.97, NFI = 0.92, IFI = 0.93, CFI = 0.93, RMSEA = 0.072), with all factor loadings ranging from 0.55 to 0.87. The AVE values for intrinsic motivation (0.62), extrinsic motivation (0.56), and amotivation (0.53). Overall, these results demonstrate satisfactory reliability and validity of the scale.

### Statistical analysis

The full path model, which included basic psychological needs, exercise behavior, sports motivation, and self-efficacy, was used for the power analysis. The complete model included four degrees of freedom and needed a sample size of 829 to identify a close fit with an RMSEA value of 0.06 and 80% statistical power (Kim et al., 2015). A total of 1,056 subjects were included in this study, providing sufficient statistical power.

The data analysis for this study followed a three-step process. First, data were collected and processed using Excel software. During this step, invalid data were removed to ensure accuracy for subsequent statistical analysis. Second, preliminary descriptive statistics were conducted using SPSS 21.0 to summarize the dataset’s basic characteristics, including calculating means and standard deviations for all variables. Additionally, we performed reliability analysis and Pearson correlation analysis. Finally, we carried out a mediation analysis using structural equation model with AMOS 24.0 software. This analysis aimed to examine whether self-efficacy mediated the relationship between basic psychological needs and exercise behavior. To access model fit, we evaluated common fit indices, including the normal fit index (NFI), goodness-of-fit index (GFI), comparative fit index (CFI), and Adjusted Goodness-of-Fit Index (AGFI). For each index, values greater than 0.90 were considered indicative of acceptable model fit (Bentler, 1990). The chi-square (χ^2^) test was also employed to evaluate overall model fit (Bollen, 1989). The root mean square error of approximation (RMSEA) was used to assess the discrepancy per degree of freedom between the hypothesized model and the population covariance matrix (Hu and Bentler, 1998). An RMSEA value below 0.08 generally indicates a reasonable fit, with values lower than 0.05 suggest reflecting a close fit (Barbeau et al., 2019). Furthermore, mediation effects were calculated using a bootstrapping analysis with 5,000 random resamples to compute 95% bias-corrected bootstrap confidence interval (Kim et al., 2015).

Additionally, to enhance the quality of latent variables in structural model analysis, it is crucial to avoid estimation errors that can arise from multiple items on a unidimensional scale. One effective strategy to achieving this is to use item parceling from single instruments as multiple indicators of the construct under consideration (Sass and Smith, 2006; Shrout and Bolger, 2002). This approach is sometimes utilized when researchers aim to understand the relationship between latent variables (Little et al., 2002). Recent literatures on item parceling strategies emphasize the importance of ensuring that the items are both unidimensional and homogeneous when utilizing this approach (Marsh et al., 2013). When those requirements for the use of the item parceling are met, the quality of indicators and model fit was quite acceptable (Koufteros et al., 2009). Additionally, research has shown that parceling three indicators is generally superior to using four or six, or the use of individual items (Bandalos, 2002). In this study, a factorial algorithm, also known as item-to-construct balance (Little et al., 2002), was employed to form the parcels. Based on the parceling procedure outlined Russell et al. (1998), we will generate three parcels for self-efficacy latent variable. In particular, an exploratory factor analysis was first conducted using the maximum likelihood method with a single factor extraction to obtain the absolute value of the factor loading for all items; Next, items were ranked based on their absolute value of the factor loading and successively assigned the items from highest to lowest factor loading to one of three parcels, with the goal of equalizing the average loadings of each parcel on its respective latent factor. The items with each parcel were then averaged to arrive at a total parcel score; finally, these parcels were used to estimate their respective latent variable within the overall latent variable (Dakanalis et al., 2015). By implementing this methodology, intergroup balance was achieved and differences between parcels were minimized, which ultimately helped to ensure the accuracy and reliability of the study results.

## Results

### Common method variance (CMV) bias

In this study, it was imperative to address the potential issue of CMV bias before data analysis because data was collected from only the same source (Podsakoff et al., 2003). We performed Harman’s single-factor test to determine CMV bias (Malhotra et al., 2017). All variables were entered into an exploratory factor analysis, and the results showed that the first unrotated factor accounted for 21.80% of the total variance, which is well below the 50% threshold value (Chaubey et al., 2019), indicating that the influence of common method bias is minimal.

### Descriptive statistics and correlation analysis

Table 1 presents the standard deviation, mean, Pearson correlation coefficient, and corresponding 95% confidence intervals (CIs) for correlations among exercise behavior, basic psychological needs, sports motivation and self-efficacy. With the exception of certain correlations related to sex and age, all bivariate correlations were positive, and their 95% CIs did not include 0, indicating small-to-moderate effect sizes.

**Table 1:** Descriptive statistics and Person correlation analysis among exercise behavior, basic psychological needs, and self-efficacy variables (*N* = 1,056).

Variables	M ± SD	1	2	3	4	5	6
1. Sex	—	1	—	—	—	—	—
2. Age	19.36 ± 1.07	—	1	—	—	—	—
3. Exercise behavior	25.02 ± 21.06	−0.08**	0.07*	1	—	—	—
		95%CI [−0.14, −0.02]	95% CI [0.01, 0.13]				
4. Basic psychological needs	62.06 ± 13.43	0.03	0.04	0.29***	1	—	—
		95%CI [−0.03, 0.09]	95% CI [−0.02, 0.10]	95% CI [0.24, 0.35]			
5. Self-efficacy	25.67 ± 5.57	−0.11***	0.03	0.33***	0.53***	1	—
		95%CI [−0.17, −0.05]	95% CI [−0.04, 0.09]	95% CI [0.27, 0.38]	95% CI [0.48, 0.57]		
6. Sports motivation	134.98 ± 26.04	−0.01	0.03	0.32***	0.70***	0.48***	1
		95%CI [−0.07, 0.06]	95% CI [−0.03, 0.10]	95% CI [0.26, 0.37]	95% CI [0.66, 0.72]	95%CI [0.43, 0.53]	

*p < 0.05, **p < 0.01, ***p < 0.001.

### Mediation analysis

**Figure 1:**
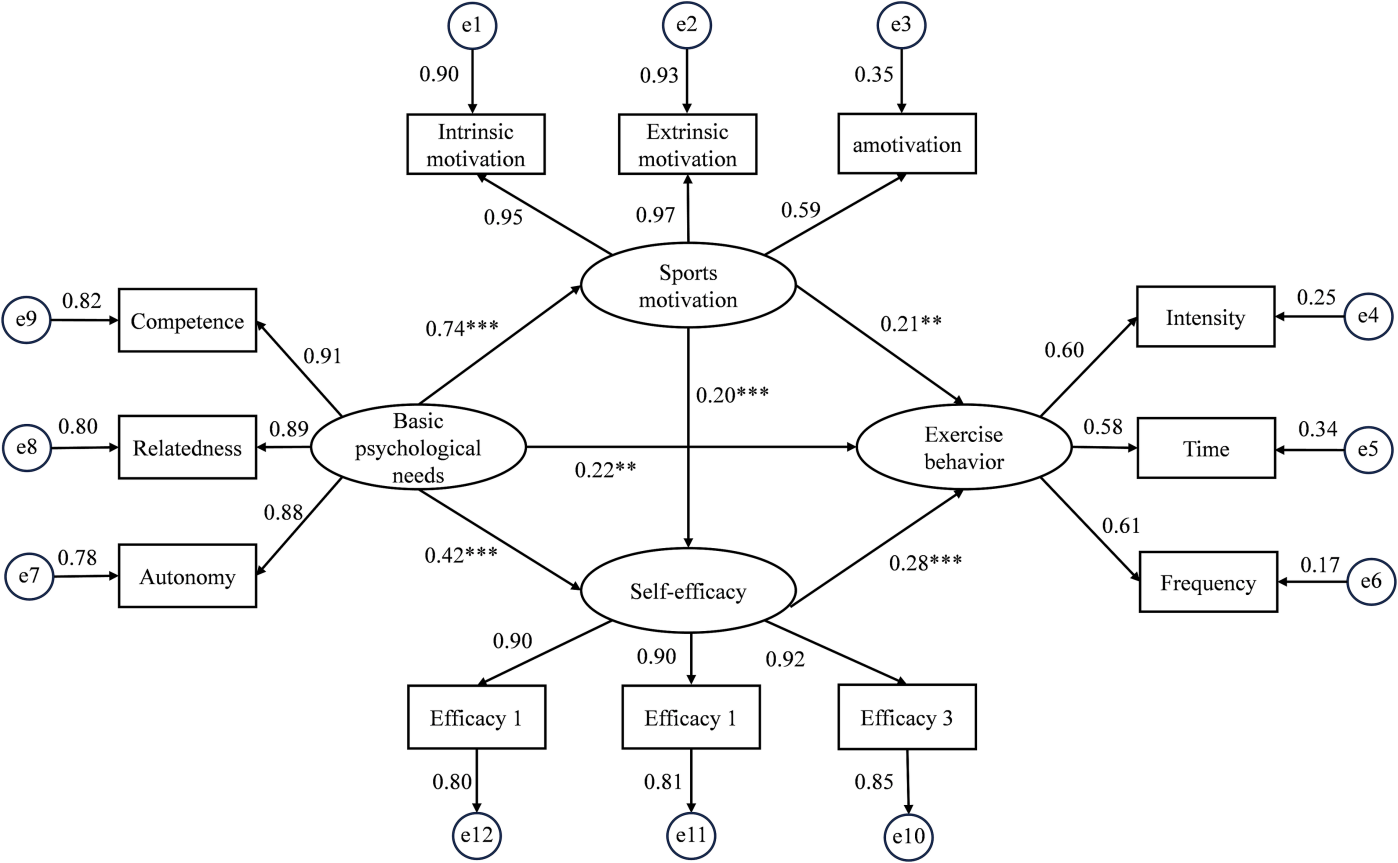
A structural model for basic psychological needs, self-efficacy, sports motivation, and exercise behavior.

Based on the principle of item parceling, we generate three parcels, Efficacy 1, Efficacy 2 and Efficacy 3, to predict self-efficacy. It is noteworthy that these parcels demonstrate high levels of internal consistency, as indicated by their corresponding alpha values of 0.849, 0.813, and 0.808, respectively. The structural equation mode was established using Amos software to further explore the influence of basic psychological needs on exercise behavior. The model’s fitting indices perform very well, with χ^2^/df = 5.149, GFI = 0.962, CFI = 0.978, AGFI = 0.938, NFI = 0.972, IFI = 0.978, RMSEA = 0.063. Figure 1 presents the direct path estimates. Independent of sex, the structural model showed significant path coefficients for basic psychological needs on exercise behavior (*β* = 0.21, *p* = 0.007, 95% CI [0.05–0.38]), which supported H1. Similarly, basic psychological needs had a significant positive association with self-efficacy (*β* = 0.42, *p* < 0.001, 95% CI [0.31–0.52]) and sports motivation (*β* = 0.75, *p* < 0.001, 95% CI [0.69–0.79]). There were also significant direct paths from sports motivation to exercise behavior (*β* = 0.21, *p* = 0.004, 95% CI [0. 07–0.36]), and from self-efficacy to exercise behavior (*β* = 0.28, *p* < 0.001, 95% CI [0.18–0.39]). Additionally, sports motivation had a significant positive association with self-efficacy (*β* = 0.20, *p* < 0.001, 95% CI [0.10–0.30]).

Next, the bootstrap method was employed to test the mediation effect within the structure model, as shown in Table 2. The results showed that there was a significant mediation effect for basic psychological needs on exercise behavior via sports motivation (*β* = 0.16, *p* = 0.004, 95% CI [0.05–0.27]) and self-efficacy (*β* = 0.12, *p* < 0.001, 95% CI [0.07–0.18]), respectively. Thus, H2 and H3 were supported. There was a significant indirect pathway for basic psychological needs on exercise behavior through sports motivation and self-efficacy in a serial fashion (*β* = 0.02, *p* = 0.002, 95% CI [0.01–0.03]) and supporting H4. Additionally, the total effect of basic psychological needs on exercise behavior was 0.52. The direct effect thus accounted for 42.31% of the total effect, while the mediation effect contributed 57.69%.

**Table 2:** Total, direct and indirect effects between basic psychological needs and exercise behavior (*N* = 1,056).

Relationship	β	SE	*p*	Bias-Corrected 95%CI	
				Upper limit	Lower limit
Total effect					
Basic psychological needs→ Exercise behavior	0.52	0.05	0.005	0.41	0.61
Direct effect					
Basic psychological needs→ Exercise behavior	0.22	0.08	0.006	0.05	0.38
Indirect effect					
Basic psychological needs→ Sports motivation→ Exercise behavior	0.16	0.06	0.004	0.05	0.27
Basic psychological needs→ Self-efficacy→ Exercise behavior	0.12	0.03	0.000	0.07	0.18
Sports motivation→ Sports motivation→ Self-efficacy→ Exercise behavior	0.02	0.01	0.002	0.01	0.03

## Discussion

The current study sought to investigate the associations between basic psychological needs and exercise behavior among college students, including the potential mediating roles of self-efficacy and sports motivation within these associations. Our findings reveal that the satisfaction of basic psychological needs is associated with exercise behavior and is also associated with exercise behavior through three distinct potential mediating pathways. First, the fulfillment of basic psychological needs is positively associated with self-efficacy, which is, in turn associated with greater participation in physical activity. Second, it is positively associated with sports motivation, which is further associated with increased exercise engagement. Finally, a sequential pathway was observed whereby sports motivation is associated with subsequent self-efficacy, which is, in turn, associated with greater exercise engagement.

The association between basic psychological needs in exercise and exercise behavior is consistent with findings reported by Gallant et al. (2024), who demonstrated that satisfaction of basic psychological needs is significantly associated with exercise behavior. According to SDT, when basic psychological needs is met, individuals are more likely to pursue physical activities that resonate with their interests, and is positively linked to sustained behavioral commitment (Ryan and Deci, 2000b). Moreover, as noted by Turner and Davis (2019), satisfaction of basic psychological needs in physical education further motivates exercise participation intentions and the development of sustained, regular exercise habits. Complementing these insights, Sylvester et al. (2018) found that higher levels of need satisfaction are directly linked to increased physical activity engagement. Indeed, satisfaction of these needs in physical activity is associated with higher levels of exercise motivation, which is, in turn, associated with a greater willingness to engage in and maintain physical activity over time. Overall, basic psychological needs are positively associated with exercise behavior for college students.

As expected, the results offer further evidence for the mediating role of self-efficacy in linking basic psychological needs to exercise behavior. Extending the findings of Li et al. (2024), our research demonstrates that the satisfaction of basic psychological needs is associated with higher levels of self-efficacy, which is, in turn, associated with more consistent exercise engagement. According to SDT, fulfilling the needs for autonomy, competence, and relatedness not only promotes adaptive behaviors but also fosters positive psychological outcomes (Ryan and Deci, 2000a). When individuals’ basic psychological needs are adequately satisfied, they possess the psychological nutrients necessary to experience a greater breadth and depth of positive psychological outcomes (Sylvester et al., 2018). Consequently, students’ perception of basic psychological needs satisfaction is positively correlated with their levels of self-efficacy. Moreover, an individual’s self-efficacy plays a crucial role in shaping their behavioral choices and determining their level of persistence (Bandura, 2014). Specifically, those with a higher degree of self-efficacy are more inclined to establish exercise goals and reach them through continuous effort (De La Cruz et al., 2021). This self-belief serves to reinforce an individual’s positive attitude toward exercise, which in turn is related to a higher frequency of exercise behaviors. Above all, students who report higher satisfaction of psychological needs tend to report greater self-efficacy, which is associated with a greater willingness to engage in and sustain exercise behavior.

Similarly, the results of this study demonstrate that basic psychological needs significantly associated with sports motivation, which is, in turn, associated with exercise behavior among college students. This finding aligns closely with prior research highlighting the role of psychological needs in relation to motivation within physical activity contexts, which is further associated with sustained engagement in exercise (Fernandez-Rio et al., 2022). On the one hand, SDT posits that motivation is influenced by individuals’ basic psychological needs (Kang et al., 2020) Specifically, when students perceive autonomy in their exercise choices, feel competent in their physical abilities, and experience social connectedness in the exercise environment, they are more likely to develop a strong motivation to engage in sports, they are more likely to be motivated to engage in sports (Hancox et al., 2018). On the other hand, students with higher sports motivation are more inclined to seek out exercise opportunities, allocate time and effort to physical activities, and maintain consistent exercise routines (Ennis, 2017). Prior research has shown that individuals who adhere to long-term exercise habits report higher levels of sports motivation (Hopkins et al., 2022), and such motivation tends to increase following the initiation of an exercise program (Moustaka et al., 2012). The current study further underscores the mediating role of sports motivation in the relationship between basic psychological needs and exercise behavior, thereby offering empirical support for their foundational role in motivational frameworks.

Consistent with previous research (Zhang et al., 2022; Zhao et al., 2023), our study also reveals that self-efficacy is positively associated with students’ exercise behaviors. In particular, individuals with higher self-efficacy are more likely to set higher exercise goals and reach them through continuous effort (De La Cruz et al., 2021). This belief reinforces the individual’s positive attitude toward exercise, which increases the frequency of exercise behavior (Qiu and Zhang, 2020). This serial mediation reveals the dynamic process from psychological need satisfaction to behavioral transformation: need satisfaction activates motivation, motivation strengthens efficacy beliefs, and efficacy ultimately is linked to behavioral implementation. This model not only deepens the understanding of the mechanism of exercise behavior, but also provides new ideas for intervention strategies, such as creating a supportive environment, stimulating sports motivation, and enhancing efficacy beliefs through multidimensional interventions, which can effectively enhance the continuity of exercise participation.

From an applied perspective, these findings indicate that students are more likely to engage in exercise behavior when their basic psychological needs were satisfied in the exercise context. Thus, teachers, parents, and school administrators should prioritize cultivating environments that respects college students’ autonomy, provides opportunities for skill development to enhance their competence, and promotes social interaction among college students. For example, physical education programs that grant students greater agency in selecting activities, deliver constructive feedback on performance, and integrate collaborative or team-based elements may enhance adherence to regular exercise habits. Instructors and curriculum designers could implement evidence-based strategies to build self-efficacy, such as setting achievable goals and providing positive reinforcement. Such interventions not only improve physical health outcomes but also contribute to students’ overall well-being by nurturing feelings of achievement and social connectedness.

This study has several limitations that should be acknowledged. First, its cross-sectional design prevents causal inference, as all variables were measured at one time point. Longitudinal or experimental studies are required to establish temporal ordering and clarify these dynamic relationships. Second, this study adopted composite total scores for basic psychological needs and sport motivation to ensure model parsimony and focus on the overall chain mediation effect. This approach, however, limited the examination of differential effects across sub-dimensions such as competence, relatedness, sports motivation. Future studies are recommended to analyze these constructs at the subscale level to explore more specific and nuanced relationships. Third, motivation was assessed using a global composite score. Although this allows for a focused investigation of overall motivation, it does not capture the unique characteristics of intrinsic motivation, extrinsic motivation, and amotivation as separate constructs. This aggregated approach may limit interpretive depth and could obscure meaningful differences between motivational subdimensions. Subsequent studies may wish to analyze these components separately to provide a more nuanced understanding of motivational processes. Finally, self-efficacy may act as an antecedent of motivation or form reciprocal relations with motivational processes. However, the current study only examined the serial mediation pathway from sports motivation to self-efficacy and then to exercise behavior, without considering the reverse effect of self-efficacy on motivation or their reciprocal associations. Future research could adopt cross-lagged or longitudinal designs to test potential reverse pathways and dynamic relationships among these variables, which would help clarify the underlying mechanisms more comprehensively.

## Conclusion

Taken together, this study suggests that fulfillment of basic psychological needs in exercise setting is positively associated with greater exercise behavior, and that sports motivation and self-efficacy as intervening variables in this cross-sectional relationship. The findings further reveal that the association between basic psychological needs and exercise behavior can be partly accounted for by the sequential associative pattern involving sports motivation and self-efficacy. These results provide preliminary conceptual support for integrating basic psychological need-supportive principles into college students’ exercise engagement, and highlight the value of theory-driven approaches targeting psychological mechanisms in this population. Promoting sports motivation and self-efficacy may be associated with more regular exercise engagement among college students. Given the cross-sectional design, longitudinal and experimental studies are warranted to verify causal pathways and temporal ordering of these associations.

## Data Availability

The datasets presented in this study can be found in online repositories. The names of the repository/repositories and accession number(s) can be found in the article/supplementary material.
